# Placental Alterations in a Chikungunya-Virus-Infected Pregnant Woman: A Case Report

**DOI:** 10.3390/microorganisms10050872

**Published:** 2022-04-22

**Authors:** Natália Salomão, Luciana Araújo, Kíssila Rabelo, Elyzabeth Avvad-Portari, Luiz de Souza, Regina Fernandes, Nathália Valle, Luiz Ferreira, Carlos Basílio-de-Oliveira, Rodrigo Basílio-de-Oliveira, Thiara de Souza, Priscila Nunes, Jorge Carvalho, Flavia dos Santos, Marciano Paes

**Affiliations:** 1 Laboratório Interdisciplinar de Pesquisas Médicas, Instituto Oswaldo Cruz, Fundação Oswaldo Cruz, Rio de Janeiro 21040-900, Brazil; 2 Departamento de Anatomia Patológica, Universidade Federal do Estado do Rio de Janeiro, Rio de Janeiro 20270-004, Brazil; lufearaujo@uol.com.br (L.A.); basiliopatologia@br.inter.net (C.B.-d.-O.); rodrigopboliveira@gmail.com (R.B.-d.-O.); 3 Laboratório de Ultraestrutura e Biologia Tecidual, Universidade do Estado do Rio de Janeiro, Rio de Janeiro 20551-030, Brazil; kissilarabelo91@gmail.com (K.R.); jjcarv@gmail.com (J.C.); 4 Departamento de Anatomia Patológica, Instituto da Mulher e da Criança Fernandes Figueira, Fundação Oswaldo Cruz, Rio de Janeiro 22250-020, Brazil; bethavvad@gmail.com; 5 Faculdade de Medicina de Campos, Campos dos Goytacazes 28035-581, Brazil; luizjosedes@gmail.com (L.d.S.); reg.fernandes@bol.com.br (R.F.); 6 Laboratório de Biotecnologia, Universidade Estadual do Norte Fluminense, Campos dos Goytacazes 28013-602, Brazil; 7 Departamento de Clínica Médica, Hospital Central da Polícia Militar, Rio de Janeiro 20211-270, Brazil; nathalia_fpv@hotmail.com; 8 Departamento de Anatomia Patológica, Instituto Nacional de Infectologia Evandro Chagas, Fundação Oswaldo Cruz, Rio de Janeiro 21040-360, Brazil; luiz.ferreira@ini.fiocruz.br; 9 Laboratório de Imunologia Viral, Instituto Oswaldo Cruz, Fundação Oswaldo Cruz, Rio de Janeiro 21040-900, Brazil; thiara.biomed@gmail.com (T.d.S.); flaviab@ioc.fiocruz.br (F.d.S.); 10 Departamento de Superintendência de Informações Estratégicas de Vigilância em Saúde, Secretaria Estadual de Saúde do Rio de Janeiro, Rio de Janeiro 20031-142, Brazil; priscila.nunes87@gmail.com

**Keywords:** chikungunya, placenta, histopathology, immunohistochemistry, electron microscopy

## Abstract

Chikungunya virus (CHIKV) is an arthropod-borne virus first isolated in Tanzania, Africa. The virus has spread to Asia as well as South and Central America through infected *Aedes* mosquitoes. Vertical transmission may also occur, and was first documented during a chikungunya outbreak in La Réunion Island in 2005. Since then, some authors have been discussing the role of the placenta in maternal–fetal CHIKV transmission. CHIKV infection is characterized by fever, headache, rash, and arthralgia. However, atypical manifestations and clinical complications, including neurological, cardiac, renal, ocular, and dermal, may occur in some cases. In this report, we describe the case of a pregnant woman infected by CHIKV during the third trimester of gestation, who presented with severe dermatological manifestations during the epidemic in Rio de Janeiro, Brazil in 2019. CHIKV RNA and antigens were detected in the placental tissue, which presented with histopathological (deciduitis, fibrin deposition, edema, fetal vessel thickening, and chorioamnionitis) and ultrastructural alterations (cytotrophoblast with mitochondrial swelling and dilated cisterns in endoplasmic reticulum, vesicles in syncytiotrophoblasts, and thickening of the basement membrane of the endothelium).

## 1. Introduction

Chikungunya virus (CHIKV) is a double-strand RNA virus belonging to the *Togaviridae* family and *Alphavirus* genus [1]. Its first identification occurred in Tanzania (Africa) in 1953, but CHIKV emerged and re-emerged in east, central, and south Africa for several years. Currently, the virus has spread to 33 countries in the world [2]. The virus’s name originates from the Makonde language meaning “that which bends up”, which refers to the intense polyarthralgia in infected individuals [3]. Mostly, the transmission occurs through bites from infected *Aedes aegypti* and *Aedes albopictus.* Then, CHIKV infects resident cells from the skin and nearby lymph nodes, or reaches directly into the bloodstream, where it disseminates to several organs such as the spleen, muscles, liver, joints, and brain [4,5]. After the incubation period (2–4 days), the patient develops symptoms such as fever, headache, myalgia, and rash in the acute phase of CHIKV infection, which lasts around 21 days [6]. However, some individuals may develop atypical neurological, cardiac, renal, ocular, and dermal manifestations. In the latter, generalized erythematous maculopapular rash, pustular rash with aphthae, vesicles, and various patterns of pigmentation are reported [7,8,9]. Moreover, in the chronic phase, the arthralgia can persist and may mimic rheumatoid arthritis, lasting for months or years [10].

CHIKV is often transmitted through the bite of infected *Aedes aegypti* and *Aedes albopictus*; however, other transmission pathways have been described, such as parenteral and vertical transmission [2,11,12]. Although rare, CHIKV can infect fetuses and newborns from CHIKV-infected women, causing significant morbidity and death; mainly, the infection occurs during the intrapartum period [13,14]. Gupta et al. found pregnancy complications such as preterm delivery, premature rupture of membranes, decreased fetal movements, intrauterine death, oligohydramnios, and preterm labor pains [15]. Cases of sepsis were reported, where the patients with CHIKV infection needed intensive-care-unit treatment, mainly in the third trimester [16]. Symptoms, such as fever, irritability, rash, edema of the limbs, meningoencephalitis, thrombocytopenia, and bullous dermatitis, as well as cardiac, renal, respiratory, and hepatic abnormalities, usually appear from the third to seventh day of life, and have been noted in babies from CHIKV-infected women [17,18]. Infection by other arboviruses, such as dengue [19], zika [20], and yellow fever [21], during pregnancy was also associated with vertical transmission, central nervous system abnormalities, risk of miscarriage, fetal death, and prematurity [22]. There are some gaps in understanding the vertical transmission of CHIKV and the role of the placenta in this type of transmission or on possible outcomes for the pregnancy, fetus, and/or newborn [23,24,25]. Based on these thoughts, we describe the placental alterations from a pregnant woman who became infected with CHIKV during the third trimester of gestation and presented with severe dermatological complications.

## 2. Materials and Methods

### 2.1. Case Description

A 32-year-old, pregnant woman (G2P1A0), at 34 weeks and 4 days of gestation, was admitted to the Hospital Plantadores de Cana, Campos dos Goytacazes, RJ, Brazil, during the 2019 chikungunya epidemic, reporting arthralgia, fever, and rash four days before, seeking assistance and further investigation. She was in good general condition: pallid (++/4+), eupneic, anicteric and acyanotic, cardiovascular system without alterations, blood pressure 120 × 80 mmHg, heart rate 96 beats/minute, with a gravid abdomen that was flaccid and without pain, single fetus with a longitudinal situation and cephalic presentation, normal uterine tone, fetal movements present, fetal heart beats 164 beats/minute, and lower limbs were swollen symmetrically +++/++++ with the presence of peripheral pulses and free calf. In the lower limbs, erythematous lesions were evidenced, which disappeared when pressed with fingers. The lesions were circumscribed and did not itch. The woman was admitted to the hospital for assistance and further investigation. 

Laboratory tests revealed anemia, a platelet count of 140,000/mm^3^, and erythrocyte sedimentation rate at 35 mm/h. The urine test revealed 23 pyocites/field. Antibiotic therapy was implemented with intravenous administration of cefazolin on the second day of hospitalization. A doppler obstetric ultrasound revealed mild oligohydramnios. 

On the third day of hospitalization, arthralgia persisted in the lower limbs; in the left lower limb, a bullous lesion appeared with local inflammation signs. The physicians requested investigation for chikungunya and a lesion evaluation. Bullous erysipelas was investigated and cefazolin administration was replaced with oxacillin. Blood test analysis revealed 5990 leukocytes/mm^3^, 145,000 platelets/mm^3^, transaminases SGOT of 39 U/L and SGTP of 23 U/L, and c-reactive protein of 55.9/mm^3^, suggesting a viral, not a bacterial, infection.

On the tenth day of hospitalization and fifth day of oxacillin, the edema evolved to 4+/4+ without lesion improvement after antibiotic therapy. The patient presented a positive anti-CHIKV IgM result and the suspicion was cellulitis resulting from CHIKV infection. The oxacillin was replaced with prednisone at 20 mg/day. Two days after the corticosteroid therapy, the lesion improved. A puncture of the bubble fluid was performed. No CHIKV RNA was detected by an RT-qPCR test on the fluid; however, a positive CHIKV RNA detection was observed in the skin lesion biopsy. After this period, the prednisone dosage was reduced to 10 mg/day. On the twenty-first day of hospitalization, the patient was discharged, at 37 weeks and 3 days of gestation. 

At 40 weeks of pregnancy, the woman returned for delivery and the placenta was collected for analysis. The newborn received care and exhibited vitality, weighing 4060 g (large for the gestational age), however evolved with increased respiratory rate, requiring ventilatory support. After 1 h, the clinical condition normalized and the baby was referred to the Join Accommodation. Afterward, the baby had a fever and hypoglycemia, requiring glucose administration. A blood test revealed 32,230/mm^3^ leukocytes, an increase in neutrophil and lymphocytes, hemoglobin of 13.5 g/dL, hematocrit of 36.7%, and 255,000 platelets/mm^3^. The serology of the newborn was negative (anti-CHIKV IgM) and no alterations in the eye and heart were observed. Real-time PCR was not performed on the baby. After 7 days of hospitalization, both mother and baby were discharged from the hospital. The delivery, specialized care, and sample collections were performed in the Plantadores de Cana Hospital. It is important to highlight that serology for dengue, zika, syphilis, and HIV (mother and baby) were performed, with negative results. A timeline of the case description is in the Supplementary Materials.

### 2.2. Viral RNA Extraction and RT-qPCR—Chikungunya Virus

The viral RNA extraction from the formalin-fixed paraffin-embedded (FFPE) placenta was performed using a PureLink^TM^ FFPE RNA Isolation Kit (Invitrogen, Waltham, MA, USA) following the manufacturer’s instructions and stored at −70 °C. CHIKV detection was performed by RT-qPCR as previously described [26] in an ABI Prism^®^ 7500 Sequence Detection System (Applied Biosystems, Foster City, CA, USA).

### 2.3. Histopathological Analysis

Placental tissues were collected and fixed in buffered formalin (10%). Smaller fragments were dehydrated in ethanol, clarified in xylene, and immersed in paraffin resin. Tissue sections (5 µm) were obtained with a microtome. Next, tissues were deparaffinized in xylene and rehydrated with decreasing concentrations of ethanol (100% to 70%). Finally, a hematoxylin and eosin (HE) stain was performed and visualized with an Olympus DP73 light microscope (Olympus, Center Valley, PA, USA). Digital images were obtained using Olympus cell Sens Standard (Olympus, Center Valley, PA, USA).

### 2.4. Immunohistochemical Procedure

Placental tissues (4 µm) were deparaffinized in xylene and rehydrated with alcohol. Antigen retrieval was performed with heat and citrate buffer, and the tissue was blocked for endogenous peroxidase with 3% hydrogen peroxidase in methanol and rinsed with Tris–HCl (pH 7.4). In order to reduce nonspecific binding, the tissues were incubated in Protein Blocker solution (Spring Bioscience, Pleasanton, CA, USA) for 10 min at room temperature. Placental tissues were incubated overnight at 4 °C with polyclonal anti-CHIKV mouse hyperimmune ascites fluids (diluted 1:700). On the following day, the tissues were incubated with a secondary complement (REVEAL complement, Spring Bioscience, Pleasanton, CA, USA) for 10 min and with a rabbit anti-mouse IgG-HRP conjugate (REVEAL polyvalent HRP, Spring Bioscience, Pleasanton, CA, USA) for 15 min at room temperature. Reactions were revealed with diaminobenzidine (Spring Bioscience, Pleasanton, CA, USA), a chromogen, and the sections were counterstained with Harris hematoxylin (Wcor Corantes, Guarulhos, SP, Brazil).

### 2.5. Quantification of CD163+ Cells by Immunohistochemistry

Slides of placental tissues (CHIKV-infected and control) were analyzed using an Olympus BX 53F microscope (Olympus, Center Valley, PA, USA). Twenty images (fields) were randomly acquired at 1000× magnification using Olympus cell Sens Standard software (Olympus, Center Valley, PA, USA). Positive cells were quantified in each of the 20 fields and the median positive cell number was determined. All analyses were accomplished in a blind test without prior knowledge of the studied groups.

### 2.6. Electron Microscopy Assay

Placental issues were prefixed with 2.5% glutaraldehyde in a sodium cacodylate buffer (0.1 M, pH 7.2) and postfixed with 1% buffered osmium tetroxide (Electron Microscopy Sciences, Hatfield, PA, USA). They were then dehydrated in an acetone series (30%, 50%, 70%, 90% and 100%; Sigma, St. Louis, MO, USA) and embedded in EPON (electron microscopy). The polymerization stage was carried out at 60 °C for 3 days. Ultrathin sections (60–90 nm) were obtained using a diamond knife (Diatome, Biel, Switzerland) adapted to a Reichert-Jung Ultracut E microtome (Markham, ON, Canada). Sections were contrasted with uranyl acetate and lead citrate (electron microscopy) and analyzed on a JEOL 1001 transmission electron microscope (JEOL, Tokyo, Japan).

### 2.7. Statistical Analyses

Data were analyzed with GraphPad prism software v 6.0 (La Jolla, CA, USA) using a Mann–Whitney statistical test. Significant differences between groups were determined considering *p* < 0.05.

## 3. Results

### 3.1. Histopathological Analysis of the Placenta

**Figure 1:**
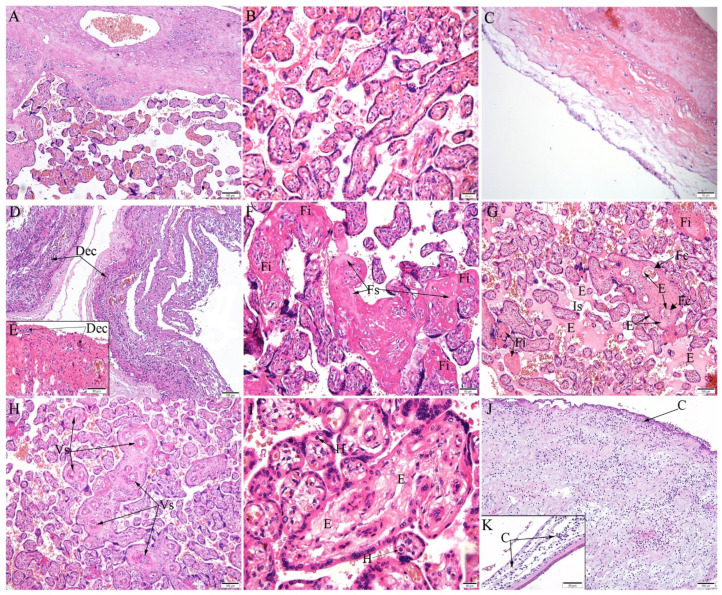
Histopathological changes in the placenta. (**A**–**C**) Control placenta (uninfected) with regular aspects: (**A**) decidua and chorionic villi, (**B**) chorionic villi, and (**C**) decidua. (**D**–**K**) CHIKV-infected placenta: (**D**–**E**) deciduitis (Dec); (**F**) fibrin deposition (Fi) and fibrosis (Fs); (**G**) edema (E) in intervillous space (Is), edema inside fetal capillaries (Fc), and fibrin deposition (Fi); (**H**) fetal vessel thickening (Vs); (**I**) edema (E) in chorionic villi, extramedullary hematopoiesis (H); (**J,K**) chorioamnionitis (C) in lower and higher magnification, respectively. Scale bar—(**A**,**D**,**G**,**H**,**J**): 100 µm; (**B**,**I**): 20 µm; (**C**,**F**,**K**): 50 µm.

The FFPE placenta was positive for CHIKV RNA after RT-qPCR. The histopathological analysis of the placenta through HE staining was performed on the maternal (decidua) and fetal portion (chorionic villi and chorionic membrane). The first exhibited mainly deciduitis (Figure 1D,E). The fetal portion showed numerous immature villi, with increases in fibrin and areas of fibrosis (Figure 1F,G). Edema was observed in the intervillous space and inside the fetal capillaries (Figure 1G). Fetal response was evidenced by extramedullary hematopoiesis (Figure 1I). In the chorionic villi, blood vessels were thick (Figure 1H) and edema in the stroma was observed (Figure 1I). In addition, the chorionic membrane presented inflammatory cells, evidencing chorioamnionitis (Figure 1J,K).

### 3.2. CHIKV Antigen Detection in the Placenta

**Figure 2:**
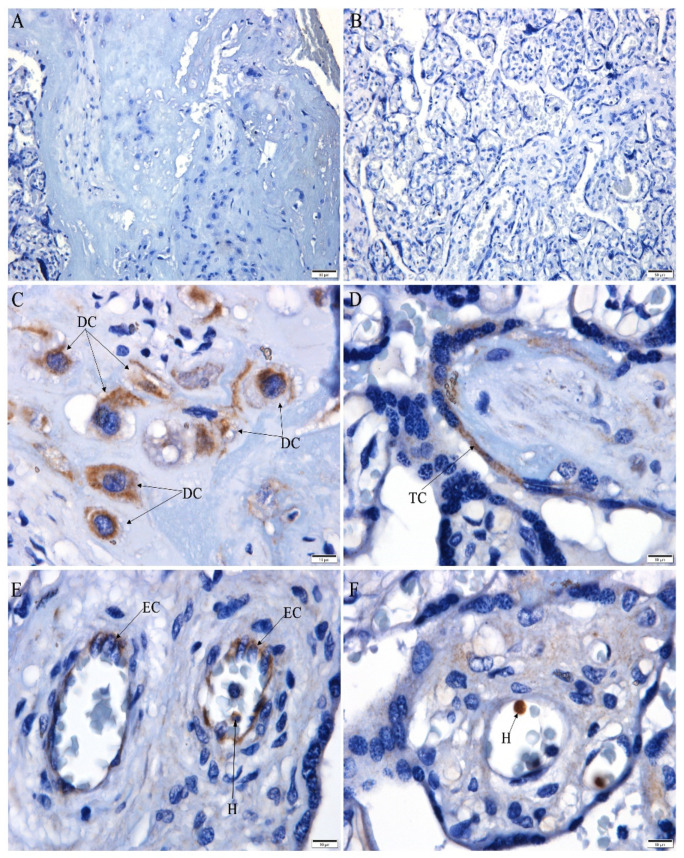
CHIKV antigen detection in the placenta. (**A**–**B**) Negative CHIKV antigen detection in the control placenta; (**C**–**F**) infected placenta with CHIKV antigen detection in: (**C**) decidual cells (DC), (**D**) trophoblast cells (TC), (**E**) endothelial cells (EC) and cell inside fetal capillary (H), and (**F**) cell inside fetal capillary (H). Scale bar—(**A**,**B**): 50 µm; (**C**–**F**): 10 µm).

Immunohistochemistry evidenced the presence of CHIKV antigens in the decidual cells (Figure 2C), trophoblast cells (Figure 2D), endothelial cells (Figure 2E), and cells inside fetal capillaries, reporting extramedullary hematopoiesis (Figure 2E,F). CHIKV antigens were not detected in the decidua (Figure 2A) or chorionic villi (Figure 2B) of the control placenta.

### 3.3. CD163 Expression in Placental Tissue

**Figure 3:**
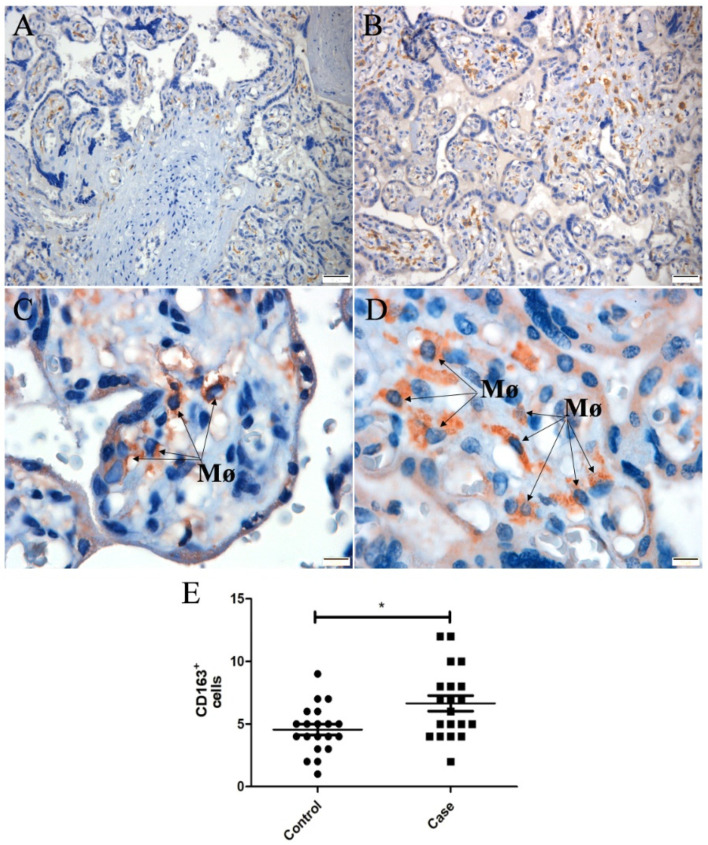
Tissue expression and quantification of CD163^+^ cells in the placenta. (**A**,**C**) Control tissue (uninfected placenta in lower and higher magnification, respectively; (**B**,**D**) CHIKV placenta in lower and higher magnification, respectively; (**E**) quantification of CD163^+^, showing increased expression in the CHIKV-infected placenta compared to the control. The square (control) and the circle (infected placenta) represent each acquired image, and the number of positive cells is represented in y axis. * indicates statistically significant differences between groups (* *p* < 0.05).

CD163 marker is used as a macrophage activation marker. In the control placenta (Figure 3A,C), CD163^+^ cells were smaller and less expressed compared to those of the CHIKV-infected placenta (Figure 3B,D). The difference in expression was confirmed by quantification (Figure 3E), which was higher in the infected placenta than in the control.

### 3.4. Ultrastructural Aspects of Placenta Infected with CHIKV

**Figure 4:**
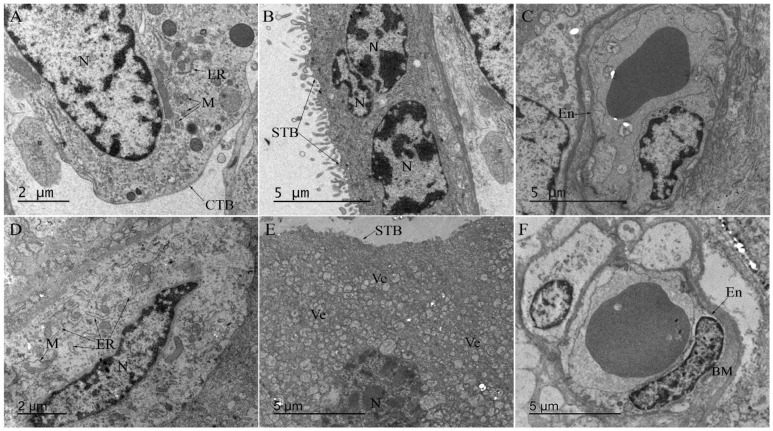
Ultrastructure of CHIKV-infected placenta. (**A**) Electron microscopy of ultrathin sections of non-CHIKV placenta with regular cytotrophoblast organelles (mitochondria and endoplasmic reticulum), (**B**) syncytiotrophoblasts, and (**C**) endothelial cells. (**D**) Mitochondrial swelling with the endoplasmic reticulum exhibiting dilated cisterns in cytotrophoblast, (**E**) vesicles in syncytiotrophoblasts, and (**F**) thickening of the basement membrane of the endothelium. CTB—Cytotrophoblast; STB—syncytiotrophoblasts; ER—endoplasmic reticulum; M—mitochondria; N—nucleus; Ve—vesicles; En—endothelium; BM—basement membrane.

The ultrastructure analysis of the CHIKV-infected placenta exhibited cytotrophoblast with mitochondrial swelling, and the endoplasmic reticulum exhibited dilated cisterns (Figure 4D), neither of which were observed in the control placenta (Figure 4A). In the syncytiotrophoblast, some vesicles were observed (Figure 4E) that were not present in the control (Figure 4B). Moreover, the thickening of the endothelial basement membrane was also observed in the infected placenta (Figure 4F) compared to the control (Figure 4C).

## 4. Discussion

*Aedes albopictus* was the vector responsible for CHIKV transmission [27] during the chikungunya epidemic on La Réunion Island between March 2005 and December 2006, where more than one-third of the population was infected; however, the first evidence of vertical transmission of CHIKV during pregnancy was also reported during that time [28]. Despite that, the role of CHIKV infection in the placenta and the consequences during pregnancy are still not well-elucidated. Severe forms of the disease have been reported in adult patients, including encephalopathy and hemorrhagic fever, mainly associated with chronic diseases or underlying conditions, such as diabetes mellitus, chronic obstructive pulmonary disease, ischemic heart disease, chronic renal failure, or alcoholic hepatopathy [29,30]. Cutaneous manifestations such as maculopapular rash during CHIKV infection have also been reported in adults and children [31,32]. 

Here, the CHIKV-infected pregnant woman exhibited an important edema and bullous lesion in the left lower limb. Although polymorphous rubelliform and roseoliform exanthema are common findings in maternal–fetal neonatal CHIKV infections [33], the vesiculobullous lesion observed in this case has also been reported [7,34] 

The newborn’s serology was negative for CHIKV anti-IgM; however, ventilatory support was necessary due to increased respiratory rate. The newborn had fever and hypoglycemia, requiring glucose administration. Healthy babies born to mothers infected with CHIKV but later presenting with fever were observed in a prospective study performed on La Réunion Island [33]. 

In order to understand the dynamic of CHIKV infection during pregnancy, the placenta was collected for analysis, as it plays an important role in respiration, nutrition, excretion, and immunity in relation to the fetus [35]. Thus, any change in these functions can cause serious consequences for the pregnancy and the baby. 

Although the pregnant woman’s serum was negative for CHIKV RNA, the placenta was positive. Considerable inflammatory infiltration was noted in the decidual region (deciduitis) and in the chorionic membrane (chorioamnionitis), possibly due to the infection response, as well as fibrin deposits, which have also been noted in ZIKV-infected placentas [36,37]. Contrastingly, calcification observed in ZIKV- and Cytomegalovirus-infected placentas was not observed here in a CHIKV-infected placenta [38]. Areas of fibrosis and edema in the chorionic villi, as well as delayed villous immaturity, were observed in a CHIKV-infected placenta [39], and elsewhere in a ZIKV-infected placenta [40]. Edema was also present in the intervillous space. In comparison, a dengue-virus-infected placenta exhibited inflammation, hemorrhage, edema, and necrosis [41]. Moreover, CHIKV antigens were detected in cells in the decidua, trophoblast, endothelium, and inside fetal capillaries, suggesting sites of entry and possible viral replication. 

The CD163 expression, an activation marker [42], was evidenced in the placental tissue and is a consistent marker of Hofbauer cells [43]. An increased number of CD163+ cells was evidenced in the CHIKV-infected placenta, resulting in hyperplasia. Moreover, these cells were larger in the CHIKV-infected placenta than in the control, evidencing activation and resulting in the production of inflammatory mediators, contributing to immunopathogenesis of the disease. In a ZIKV-infected placenta, CD163 protein was colocalized with ZIKV NS3 [44] and NS1 antigens [36], evidencing sites of viral replication. Rosenberg et al. observed increased numbers of Hofbauer cells (CD163^+^ cells) present in the stroma of all villi of a ZIKV-infected placenta [45]. By the fourth or fifth month of gestation, these cells decrease in quantity; however, hyperplasia of Hofbauer cells was reported to occur in several pathologic conditions of pregnancy, such as ascending infections, TORCH infections, and villitis of unknown etiology [46]. In this line of thinking, these cells seem to contribute to the immunopathogenesis of these infections.

The ultrastructure analysis allowed the characterization of placental cells’ organelles. The mitochondria play an important role in the metabolic function of the cell, and their interactions with the endoplasmic reticulum are critical to cell homeostasis and signaling [47]. Here, the CHIKV-infected placenta exhibited cytotrophoblast with mitochondrial swelling, and the endoplasmic reticulum exhibited dilated cisterns. Any changes in these organelles can be detrimental to the optimal functioning of the cell and, consequently, the tissue. Furthermore, mitochondrial swelling, a characteristic of apoptosis, has been reported in pre-eclamptic cases [48]. The thickening of the endothelial basement membrane can alter the absorption of gases and nutrients between the mother and the baby [39]. 

Although the baby showed clinical changes, in this work, we could not confirm vertical transmission. However, it was shown that CHIKV can infect the placenta, causing changes that may impact the pregnancy and the baby. Therefore, studies such as this play an important role in understanding CHIKV transmission in the mother–child interface.

## Data Availability

Data generated or analyzed during this study are included in this published manuscript.

## Supplementary Materials

The following are available online at https://www.mdpi.com/article/10.3390/microorganisms10050872/s1, Figure S1. Clinical case description timeline—mother and baby.
