# Supplementary figures and images for: Placental Alterations in a Chikungunya-Virus-Infected Pregnant Woman: A Case Report
Source: Microorganisms. 2022 Apr 22;10(5):872. doi: 10.3390/microorganisms10050872 (PMC9144120; doi:10.3390/microorganisms10050872)

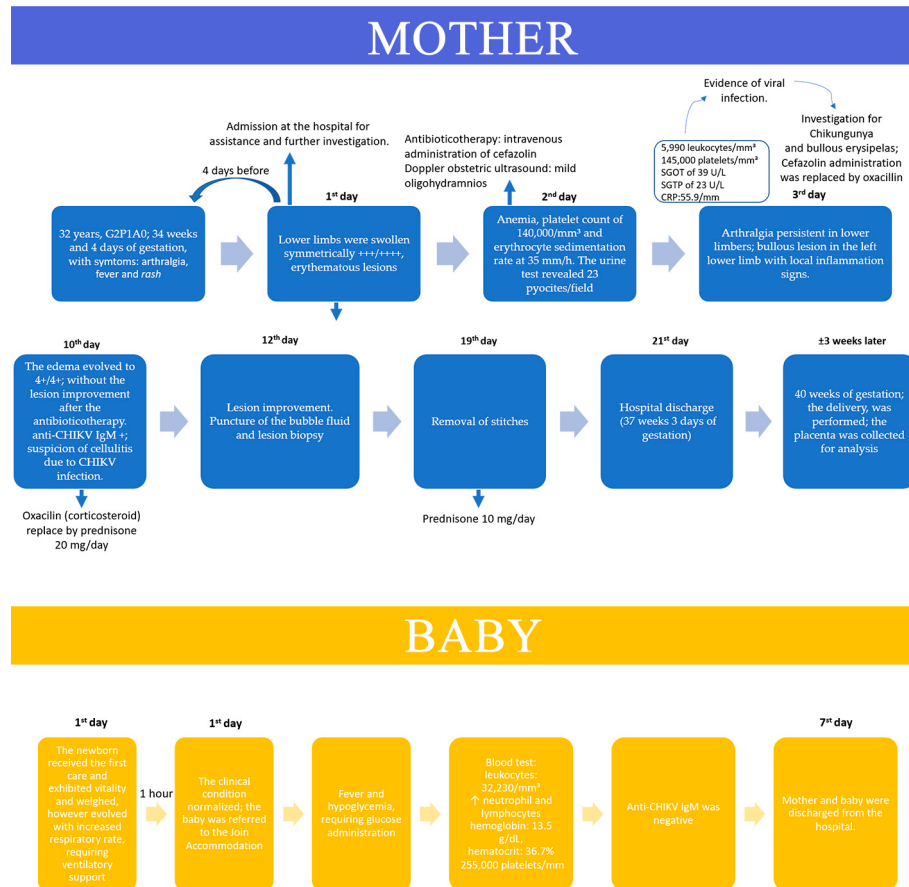

Figure S1. Clinical case description timeline—mother and baby.

Supplement: Supplementary file 1 [file microorganisms-10-00872-s001.zip › microorganisms-1624809-supplementary.pdf]
